# Emerging Tobacco-Related Cancer Risks in China: A Nationwide, Prospective Study of 0.5 Million Adults

**DOI:** 10.1002/cncr.29560

**Published:** 2015-09-01

**Authors:** Zheng-Ming Chen, Richard Peto, Andri Iona, Yu Guo, Yi-Ping Chen, Zheng Bian, Ling Yang, Wei-Yuan Zhang, Feng Lu, Jun-Shi Chen, Rory Collins, Li-Ming Li, for the China Kadoorie Biobank Collaborative Group

**Affiliations:** 1 Clinical Trial Service Unit and Epidemiological Studies Unit, Nuffield Department of Population Health, University of OxfordUnited Kingdom; 2 Chinese Academy of Medical SciencesBeijing, China; 3 Liuzhou Center for Disease Control and PreventionLiuzhou, China; 4 Zhejiang Provincial Center for Disease Control and PreventionHangzhou, China; 5 China National Center for Food Safety Risk AssessmentBeijing, China; 6 Department of Epidemiology and Biostatistics, School of Public Health, Peking UniversityBeijing, China

**Keywords:** cancer, cessation, China, cohort study, smoking

## Abstract

**BACKGROUND:**

In China, cigarette consumption has increased substantially since the 1980s, almost exclusively in men. This study was aimed at assessing the emerging cancer risks.

**METHODS:**

A nationwide, prospective study recruited 210,259 men and 302,632 women aged 30 to 79 years from 10 areas of China from 2004 to 2008; approximately 18,000 incident cancers were recorded during 7 years of follow-up. Cox regression yielded adjusted risk ratios (RRs) comparing smokers (including those who had stopped because of illness but not those who had stopped by choice) with never-smokers.

**RESULTS:**

Among men, 68% were smokers; their overall cancer risk was significantly increased (RR, 1.44; 95% confidence interval [CI], 1.37-1.53), and it was greater in urban (RR, 1.55; 95% CI, 1.41-1.70) than in rural areas (RR, 1.39; 95% CI, 1.30-1.49). This excess accounted for 23% of all cancers between the ages of 40 and 79 years, with significantly elevated risks of lung cancer (RR, 2.51; 95% CI, 2.18-2.90), liver cancer (RR, 1.32; 95% CI, 1.12-1.54), stomach cancer (RR, 1.34; 95% CI, 1.16-1.55), esophageal cancer (RR, 1.47; 95% CI, 1.24-1.73), and an aggregate of 5 other minor sites (RR, 1.52; 95% CI, 1.25-1.86). For lung cancer, the RRs were much greater for nonadenocarcinoma (RR, 5.83; 95% CI, 5.02-6.77) than for adenocarcinoma (RR, 1.78; 95% CI, 1.36-2.34). Among exsmokers (6.7%) who had stopped by choice, there was little excess cancer risk approximately 15 years after quitting. Among the few female smokers (3%), the overall cancer risk was also significantly increased (RR, 1.42; 95% CI, 1.28-1.57). Smoking was estimated to cause approximately 435,000 new cancers per year in China (approximately 360,000 in men and approximately 75,000 in women).

**CONCLUSIONS:**

In China, smoking now causes a quarter of all adult male cancers. High male uptake rates before the age of 20 years and nearly universal use of cigarettes foreshadow substantial tobacco-attributed risks in China unless there is widespread cessation. ***Cancer* 2015;121:3097-106.** © 2015 The Authors. *Cancer* published by Wiley Periodicals, Inc. on behalf of *American Cancer Society*.

## INTRODUCTION

Tobacco smoking is one of the most important avoidable causes of premature death and major disability globally.^1^–^6^ In developed countries (eg, the United States and the United Kingdom), where cigarettes have been used widely for several decades, smoking was responsible by the 1980s for approximately one-third of all deaths and more than half of all cancer deaths in middle age. Since then, the tobacco-attributed mortality rate has been declining steadily in most developed countries, but it is growing in many developing countries. China, with one-fifth of the world's population, now produces and consumes approximately 40% of the world's cigarettes, with much of the rapid increase taking place since the early 1980s, almost exclusively in men.^7^–^10^ There is evidence that the lung cancer mortality rate is rising steadily in China,^11^ but the substantial increases in cigarette smoking among Chinese men are, however, as yet too recent for the full eventual health consequences to be seen with respect to cancer and other diseases.^12^

Over the last few decades, large studies of smoking and mortality have been conducted in particular regions of China or nationwide.^13^–^18^ Nearly all individuals included in these studies were born before 1950 and, unlike smokers born in recent decades, had not smoked cigarettes persistently since early adulthood or had smoked forms of tobacco (eg, pipe tobacco and hand-rolled cigarettes) that carry a lower risk than cigarettes. Thus, their tobacco-attributed risk was much less extreme than the risk for their counterparts in the West; they had, for example, only a 2- to 4-fold increased risk of lung cancer^13^–^18^ versus the 20- to 30-fold increased risk among smokers in the West.^1^–^6^ The recent substantial increase in cigarette consumption in China can be expected eventually to cause a substantial increase in risk over the next few decades, especially among men. Large changes in diet, physical activity, indoor and outdoor air pollution, and chronic infective processes that are taking place in China may well greatly alter the background rates of particular diseases among nonsmokers and change the absolute effects of tobacco smoking.^11^,^12^

Despite the growing tobacco epidemic, there are no reliable estimates about smoking-related cancer risks in China for the current decade or about the benefits of stopping. We aim to address these issues with data from a recent nationwide, prospective study (China Kadoorie Biobank) that began in 2004-2008. By applying population-attributed fractions of cancer from smoking to 2010 national cancer incidence data, we also estimate the number of smoking-attributed cancer cases in China.

## MATERIALS AND METHODS

### Baseline Survey

Details about the China Kadoorie Biobank design, methods, and participants have been described elsewhere.^19^,^20^ Briefly, the 2004-2008 baseline survey took place in 10 geographically diverse areas of China, 4 urban and 6 rural (or semirural [Suzhou]). These areas were chosen to cover a range of socioeconomic statuses, disease patterns, likely exposures to certain risk factors, and health record system quality. All study areas are part of the China Center for Disease Control and Prevention's Disease Surveillance Points and should, in aggregate, be reasonably nationally representative (eg, the proportion living in rural areas and the smoking prevalence).^21^ All nondisabled residents aged 35 to 74 years were invited to attend local survey clinics. Trained health workers administered laptop-based questionnaires on sociodemographic status, smoking, alcohol drinking, diet, physical activities, exposure to indoor air pollution, self-reported medical history, and reproductive history (in women), and they took physical measurements (eg, height, weight, waist and hip circumferences, bio-impedance, blood pressure, heart rate, and lung function) with validated instruments and blood for long-term storage. Overall, 512,891 individuals, 210,259 men and 302,632 women, (including a few just outside the age range of 35-74 years) participated and gave written informed consent for follow-up. Prior international, national, and local ethics approval was obtained.

### Assessment of Smoking Status

The interviewer-administered questionnaire on smoking included the frequency, type, and amount of smoking both currently and in the past, the degree of inhalation, the age at which regular smoking was first started, the age at which smoking was last stopped, and the main reason for cessation. Regular smokers were defined as those who reported having ever smoked 1 or more cigarettes (or their equivalent) daily for at least 6 months. Among regular smokers who had stopped 6 or more months before recruitment, approximately half did so because of ill health, and they were still counted as smokers in the analyses; those who had stopped by choice were analyzed separately to assess the effects of cessation. To help validate exposure to smoking, exhaled carbon monoxide was also measured (CareFusion MicroCO meter).^22^

### Follow-Up for Mortality and Cancer Incidence

Each participant's vital status was obtained periodically through China's Disease Surveillance Point death registries,^21^ which were supplemented annually by checks against local residential records and health insurance claim databases and by active confirmation through street committees or village physicians/administrators. Deaths were coded with *International Classification of Diseases, Tenth Revision* (*ICD-10*) by trained staff blinded to the baseline information. Causes of death from official death certificates were supplemented, if necessary, by a review of medical records. For the small proportion of deaths (<5%) without any recent medical attention, standardized procedures were used to determine probable causes from symptoms or signs described by informants.^23^ Additional data on cancer incidence were collected through linkages with established chronic disease registries (for cancer, stroke, ischemic heart disease, and diabetes) in the study areas and with national health insurance claim databases. Nearly all of the China Kadoorie Biobank population is now covered by the health insurance system, which records details of all hospitalized events (with *ICD-10* codes) and coded examination and treatment procedures.^20^ Information on cancer histological subtypes was also collected for a subset of the cases through cancer registries or reviews of hospital medical notes as part of the ongoing outcome adjudication for major diseases.

By January 1, 2014, 25,488 participants (5%) had died after 7 years of follow-up, 2411 (0.5%) were lost to follow-up, and 18,345 had been diagnosed with their first incident cancer (*ICD-10* codes C00-C97).

### Statistical Analysis

All the analyses were performed separately for men and women and excluded the small number of participants with a prior history of cancer at the baseline. Cox regression models were used to obtain adjusted risk ratios (RRs) that compared smokers (including those who had stopped because of ill health) with never-smokers. Sex-specific analyses were stratified for the age at risk (5-year groups) and the geographical location (4 urban locations and 6 rural/semirural locations), and they were adjusted simultaneously for education (4 levels) and alcohol drinking (3 levels: never, occasional, or ever regular). RRs for particular smoking categories are presented along with the variance of the log risk in each category, so each RR has a 95% confidence interval (CI) that appropriately reflects the number of subjects and cancer cases in that smoking category.^24^ We did not categorize smoking exposure by pack-years because smoking 10 cigarettes a day for 40 years may produce very different risks late in life than smoking 20 cigarettes a day for 20 years. In addition to the amount smoked and the degree of inhalation, we also assessed the effects by the age at which smoking was first started, which, given duration, also affects the disease risk independently.^25^ The population attributable fraction (PAF) was calculated with the following equation:


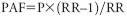


where P is the prevalence of smoking among those developing the relevant cancer in the China Kadoorie Biobank. This provides a valid PAF estimate even when there is confounding.^26^ All analyses used SAS 9.3.

## RESULTS

**Figure 1:**
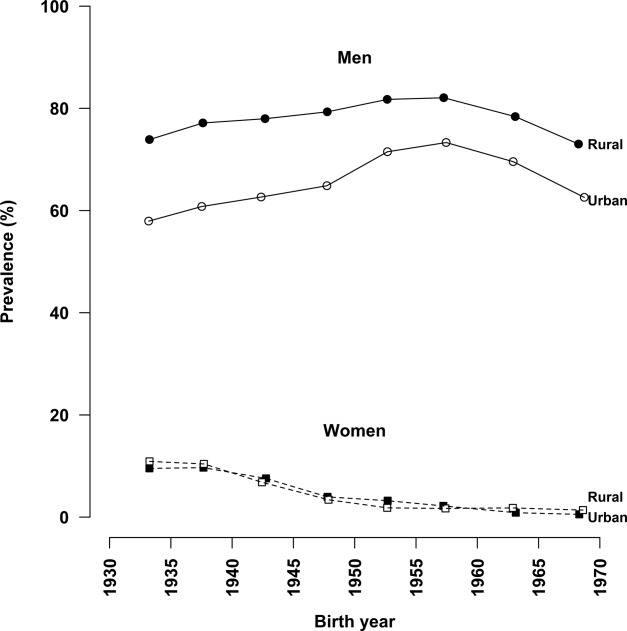
Prevalence of ever regular smoking by year of birth among men and women.

Overall, 74% of men were ever regular smokers (including 6.7% who had stopped by choice), whereas only 3% of women were. For both sexes, the mean exhaled carbon monoxide level was significantly higher for current smokers than for ex-smokers or never-smokers (Supporting Table 1 [see online supporting information]). Among men, the prevalence of ever regular smoking did not vary much by area, but overall, it was significantly higher in rural areas than urban areas (78.2% vs 66.4%, *P* <. 00001). It also varied only slightly with age or year of birth (Fig. 1), household income, and level of education (except among those educated to a tertiary level, for whom it was considerably lower). Younger smokers were more likely to have started at a younger age (approximately 20 years for men born in 1970 versus approximately 26 years for men born in 1930; overall mean age, 22 years) and to smoke cigarettes (vs other forms of tobacco) both when they first started and when they last smoked. Although the mean age of starting differed little by area, the proportion of men who smoked cigarettes when they first started or last smoked was significantly higher in urban areas than in rural areas, especially in older birth cohorts (Supporting Fig. 1 [see online supporting information]).

Among women, the prevalence of ever regular smoking was associated positively with increasing age at the baseline or an earlier birth cohort (0.7% for those born in the 1970s, 1.4% for those born in the 1960s, 2.9% for those born in the 1950s, 7.5% for those born in the 1940s, and 10.1% for those born in the 1930s; Fig. 1). Because the mean age for starting to smoke regularly among smokers was similar across different birth cohorts (approximately 27 years), this inverse trend with the year of birth reflects chiefly a progressive decline in the probability of starting to smoke among young women over the past few decades.

During approximately 1.4 million man-years of follow-up, 8566 men developed cancer between the ages of 40 and 79 years, and the risk was strongly associated with ever regular smoking (Table 1), with age- and area-standardized incidence rates of 6.7 per 1000 in smokers and 4.7 per 1000 in never-smokers. The multivariate adjusted RR for overall cancer in men was 1.44 (95% CI, 1.37-1.53) and was greater in urban areas (RR, 1.55; 95% CI, 1.41-1.70) than rural areas (RR, 1.39; 95% CI, 1.30-1.49). Across 4 urban areas, the RR for smokers ranged from 1.44 to 1.66, whereas in rural areas, it ranged from 1.24 to 1.75 (Supporting Fig. 2 [see online supporting information]). When the analyses were restricted to cancer mortality, the findings were similar (RR, 1.52; 95% CI, 1.47-1.58). If these associations with smoking are largely causal, then 23% of all male cancer cases between the ages of 40 and 79 years in the current study can be attributed directly to ever regular smoking.

**TABLE 1:** Number of Cancer Cases at the Ages of 40 to 79 Years, Adjusted RRs, and Proportions of Smoking-Attributable Risk in Men and Women

	Men				Women			
		
	No. of Events		RR (95% CI)^b^	PAF (%)^c^	No. of Events			
				
	Never-Smokers	Smokers^a^			Never-Smokers	Smokers^a^	RR (95% CI)^b^	PAF (%)^c^
Lung cancer	232	1481	2.51 (2.18-2.90)^d^	50	1031	124	2.28 (1.84-2.81)^d^	7
Liver cancer	207	774	1.32 (1.12-1.54)^e^	18	458	35	1.49 (1.01-2.20)^e^	2
Stomach cancer	250	952	1.34 (1.16-1.55)^d^	19	582	33	1.19 (0.81-1.75)	0
Esophageal cancer	179	768	1.47 (1.24-1.73)^d^	24	442	17	1.24 (0.71-2.17)	1
Breast cancer					1286	33	0.99 (0.69-1.42)	0
Five minor sites^f^	131	523	1.52 (1.25-1.86)^d^	25	421	21	1.04 (0.65-1.67)	0
All other cancer	632	1783	1.10 (1.00-1.21)	7	3786	185	1.29 (1.10-1.51)^e^	1
All cancer	1631	6281	1.44 (1.37-1.53)^d^	23	8006	448	1.42 (1.28-1.57)^e^	2

Abbreviations: CI, confidence interval; PAF, population attributed fraction; RR, risk ratio.

This table excludes 2577 participants (including 969 males) with cancer at the baseline.

^a^Smokers exclude 15,281 ever regular smokers who had stopped smoking by choice but not those who had stopped because of ill health.

^b^RRs were adjusted for area and age at risk (strata) and for education (4 levels) and alcohol use (never, occasional, or regular).

^c^The RRs that were used for calculating PAFs were for all ever regular smokers, including those who had stopped by choice.

^d^*P* <. 001.

^e^*P* <. 01.

^f^Five minor sites include: mouth, pharynx, bladder, larynx, and pancreas (*International Classification of Diseases, Tenth Revision* codes C00-C14, C25, C32, and C67).

Among smokers, there were significantly increased risks of several major cancers, including lung cancer (RR, 2.51; 95% CI, 2.18-2.90), liver cancer (RR, 1.32; 95% CI, 1.12-1.54), stomach cancer (RR, 1.34; 95% CI, 1.16-1.55), esophageal cancer (RR, 1.47; 95% CI, 1.24-1.73), and an aggregate of cancer at 5 other minor sites (RR, 1.52; 95% CI, 1.25-1.86; Table 1) with PAFs of 50%, 18%, 19%, 24%, and 25% respectively. Again, the excess risks were generally greater in urban smokers than in rural smokers (data not shown). For colorectal cancer (583 cases), no significant association with smoking was observed (RR, 1.08; 95% CI, 0.99-1.18). Compared with ever regular smokers, the relative risk estimates for current smokers versus never-smokers were similar, but the population attributable fractions were somewhat smaller, mainly because of the lower prevalence (Supporting Table 2 [see online supporting information]).

**Figure 2:**
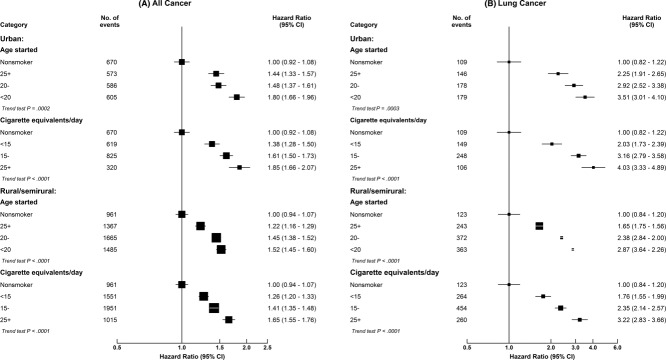
Adjusted risk ratios for (A) all cancer and (B) lung cancer by the age at which smoking was started and by the amount last smoked in urban and rural men. The adjusted risk ratios are plotted on floating absolute scales. The area of each black square is inversely proportional to the variance of the log risk ratio, which also determines the 95% confidence intervals (CIs).

Table 2 shows the adjusted RRs for all cancer and site-specific cancer among men by certain smoking characteristics. In most cases, those men who first started smoking regularly before the age of 20 years (mean age, approximately 17 years) were at greater risk than those who had begun just 4 to 5 years later. For all cancer, the observed RR for men who started at less than 20 years of age was 1.61 (95% CI, 1.54-1.69), whereas it was 1.30 (95% CI, 1.24-1.36) for those who started at ≥25 years; for lung cancer, the RRs were 3.17 (95% CI, 2.91-3.46) and 1.90 (95% CI, 1.72-2.10), respectively. Again, the excess risks were greater among urban men than rural men for a given pattern of smoking, even though the CIs overlapped in some cases (Fig. 2). Among urban male smokers who started before the age of 20 years (ie, the uptake pattern now typical among young men throughout China), most had always smoked manufactured cigarettes, and the RR for all cancer was 1.80 (95% CI, 1.66-1.96), and this suggested that at nonsmoker rates, 44% (0.80 of 1.80) of all cancer cases among men who started before the age of 20 years would have been avoided. For lung cancer, there were also significant dose-response relations with the amount smoked and the degree of inhalation (Table 2).

**TABLE 2:** Adjusted RRs for All Cancer and Site-Specific Cancer According to the Characteristics of Smoking in Men

	All Cancer		Lung Cancer		Liver Cancer		Stomach Cancer		Esophageal Cancer		5 Minor Sites		All Other Cancer	
	No. of Events	RR (95% CI)	No. of Events	RR (95% CI)	No. of Events	RR (95% CI)	No. of Events	RR (95% CI)	No. of Events	RR (95% CI)	No. of Events	RR (95% CI)	No. of Events	RR (95% CI)
Smoking category														
Never-smoker	1631	1.00 (0.95-1.05)	232	1.00 (0.88-1.14)	207	1.00 (0.87-1.15)	250	1.00 (0.88-1.14)	179	1.00 (0.86-1.16)	131	1.00 (0.84-1.19)	632	1.00 (0.92-1.08)
Exsmoker	654	1.15 (1.06-1.24)	130	1.53 (1.28-1.81)	75	1.11 (0.88-1.39)	90	1.04 (0.84-1.27)	63	1.08 (0.84-1.38)	45	0.98 (0.73-1.31)	251	1.12 (0.99-1.27)
Smoker	6281	1.44 (1.41-1.48)	1481	2.51 (2.37-2.66)	774	1.32 (1.22-1.42)	952	1.34 (1.25-1.44)	768	1.47 (1.36-1.58)	523	1.52 (1.38-1.67)	1783	1.10 (1.05-1.16)
*P* (heterogeneity)		<.0001		<.0001		.0022		<.0001		<.0001		<.0001		.1007
Age of starting smoking (mean)														
≥25 y (31.0 y)	1940	1.30 (1.24-1.36)	389	1.90 (1.72-2.10)	216	1.13 (0.99-1.30)	313	1.15 (1.03-1.29)	266	1.52 (1.34-1.71)	172	1.45 (1.24-1.69)	584	1.09 (1.00-1.18)
20-24 y (21.1 y)	2251	1.47 (1.41-1.54)	550	2.64 (2.43-2.87)	293	1.43 (1.27-1.60)	329	1.39 (1.25-1.55)	266	1.48 (1.31-1.67)	171	1.41 (1.21-1.64)	642	1.10 (1.02-1.19)
<20 y (16.8 y)	2090	1.61 (1.54-1.69)	542	3.17 (2.91-3.46)	265	1.46 (1.29-1.65)	310	1.57 (1.40-1.76)	236	1.40 (1.23-1.59)	180	1.79 (1.54-2.09)	557	1.14 (1.04-1.24)
*P* (trend)^a^		<.0001		<.0001		.0087		.0002		.3832		.0497		.4372
Always inhaled into lungs														
No	4057	1.41 (1.37-1.46)	891	2.28 (2.13-2.44)	505	1.32 (1.20-1.44)	643	1.33 (1.22-1.44)	509	1.46 (1.33-1.60)	357	1.56 (1.40-1.74)	1152	1.10 (1.04-1.17)
Yes	2224	1.51 (1.45-1.58)	590	2.91 (2.68-3.17)	269	1.37 (1.21-1.54)	309	1.38 (1.23-1.54)	259	1.48 (1.30-1.67)	166	1.47 (1.26-1.72)	631	1.11 (1.03-1.21)
*P* (heterogeneity)^a^		.0105		<.0001		.6313		.6092		.8971		.5598		.8143
Cigarette equivalents/day (mean)														
<15 (7.9)	2170	1.31 (1.25-1.37)	413	1.90 (1.72-2.10)	275	1.30 (1.15-1.46)	358	1.21 (1.09-1.35)	330	1.37 (1.23-1.54)	153	1.28 (1.09-1.50)	641	1.11 (1.03-1.20)
15-24 (19.2)	2776	1.48 (1.43-1.54)	702	2.68 (2.49-2.89)	351	1.38 (1.24-1.53)	422	1.45 (1.32-1.60)	257	1.37 (1.21-1.56)	256	1.66 (1.47-1.88)	788	1.07 (0.99-1.15)
≥25 (35.2)	1335	1.72 (1.63-1.82)	366	3.59 (3.22-3.99)	148	1.32 (1.11-1.56)	172	1.45 (1.24-1.69)	181	2.04 (1.74-2.40)	114	1.75 (1.44-2.12)	354	1.20 (1.07-1.33)
*P* (trend)^a^		<.0001		<.0001		.7518		.0258		.0004		.0092		.4336

Abbreviations: CI, confidence interval; RR, risk ratio.

This table excludes 969 participants with cancer at the baseline.

^a^The RRs are relative to nonsmokers. Heterogeneity and trend tests were calculated within smokers.

Table 3 shows the separate relation between smoking and the histological subtype of lung cancer for a subset of the cases. The excess risks were significantly greater for nonadenocarcinoma (ie, squamous cell carcinoma, small cell carcinoma, and large cell carcinoma) than for adenocarcinoma with adjusted RRs of 5.83 (95% CI, 5.02-6.77) versus 1.78 (95% CI, 1.36-2.34). For nonadenocarcinoma, there was a particularly strong dose-response relation with the amount smoked: the RRs were 3.0, 6.9, and 11.2 for those who smoked <15, 15 to 24, and 25 or more cigarettes (or their equivalent) daily (*P* <. 0001 for trend). For adenocarcinoma, no similar pattern was seen, although the number of cases involved was small.

**TABLE 3:** Adjusted RRs for Lung Cancer Histological Subtypes by the Characteristics of Smoking in Men

	Adenocarcinoma		Nonadenocarcinoma^a^		Other or Unknown Type	
	No. of Events	RR (95% CI)	No. of Events	RR (95% CI)	No. of Events	RR (95% CI)
Smoking category						
Never-smoker	18	1.00 (0.62-1.61)	16	1.00 (0.61-1.64)	8	1.00 (0.50-2.02)
Exsmoker	5	0.75 (0.31-1.80)	11	1.75 (0.97-3.16)	4	1.32 (0.49-3.51)
Smoker	61	1.78 (1.36-2.34)	215	5.83 (5.02-6.77)	64	3.74 (2.80-5.00)
*P* (heterogeneity)		.0353		<.0001		.0008
Age of starting smoking (mean)						
≥25 y (31.0 y)	21	1.66 (1.08-2.56)	54	4.36 (3.32-5.72)	13	1.83 (1.06-3.17)
20-24 y (22.3 y)	20	1.60 (1.03-2.49)	89	6.77 (5.50-8.31)	28	4.72 (3.26-6.83)
<20 y (17.0 y)	20	1.99 (1.26-3.12)	72	6.89 (5.43-8.75)	23	5.04 (3.31-7.67)
*P* (trend)^b^		.5870		.0184		.0091
Always inhaled into lungs						
No	37	1.79 (1.29-2.48)	123	5.59 (4.67-6.70)	37	3.08 (2.20-4.31)
Yes	24	1.64 (1.09-2.47)	92	6.30 (5.10-7.78)	27	4.39 (2.99-6.44)
*P* (heterogeneity)^b^		.7444		.4025		.1745
Cigarette equivalents/day (mean)						
<15 (7.7)	23	1.76 (1.16-2.66)	40	2.97 (2.16-4.07)	18	2.74 (1.71-4.38)
15-24 (19.2)	30	1.84 (1.28-2.64)	112	6.93 (5.76-8.32)	38	4.64 (3.35-6.41)
≥25 (35.2)	8	1.34 (0.67-2.70)	63	11.18 (8.68-14.40)	8	2.57 (1.27-5.21)
*P* (trend)^b^		.6504		<.0001		.6085

Abbreviations: CI, confidence interval; RR, risk ratio.

^a^Includes squamous cell carcinoma, small cell lung carcinoma, and large cell lung carcinoma.

^b^The RRs are relative to nonsmokers. Heterogeneity and trend tests were calculated within smokers.

**Figure 3:**
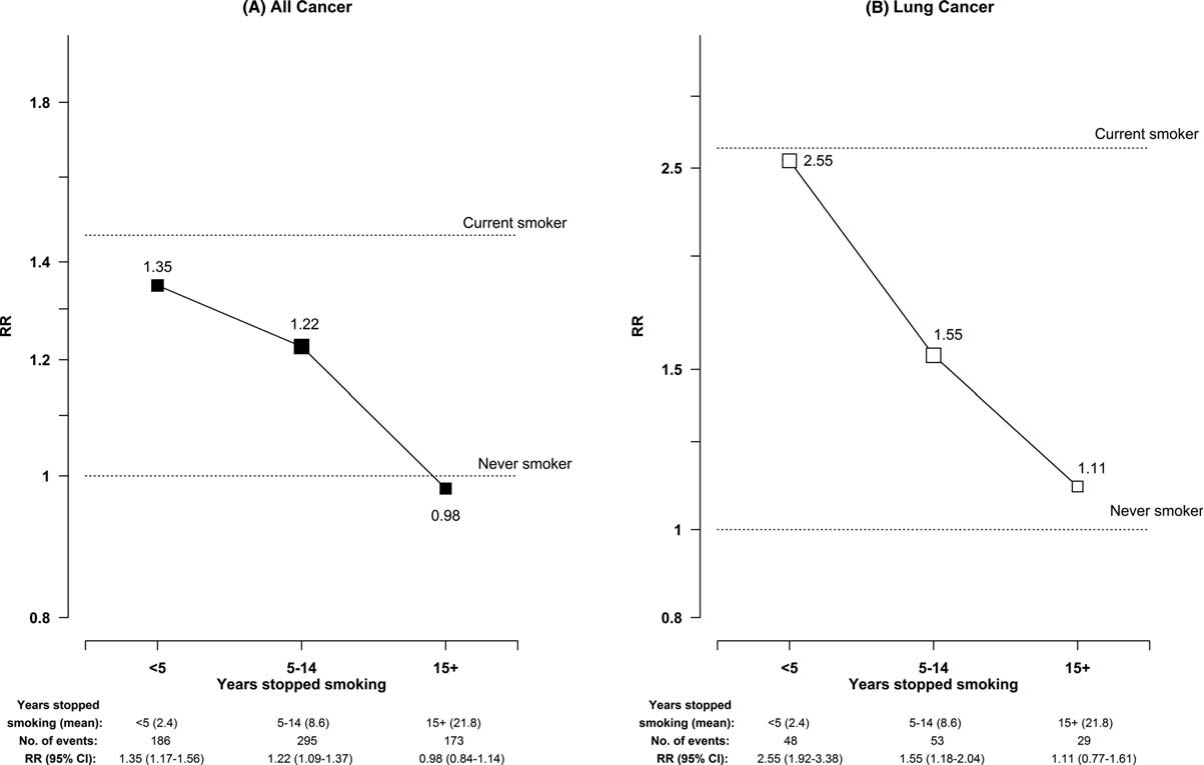
Adjusted risk ratios (RRs) for (A) all cancer and (B) lung cancer by the number of years stopped smoking among male exsmokers who had stopped by choice. The adjusted RRs are plotted on floating absolute scales. The area of each black or white square is inversely proportional to the variance of the log RR, which also determines the 95% confidence intervals (CIs).

In this study, 6.7% of the men (9% of ever regular smokers) were former smokers who had stopped by choice. Among them, the RRs were 1.15 (95% CI, 1.06-1.24) for all cancer and 1.53 (95% CI, 1.28-1.81) for lung cancer (Table 2). Figure 3 further shows the RRs by years of quitting and compares them with risks for current smokers and never-smokers. The excess risks decreased with increasing years of quitting reported at the baseline, with RRs for all cancer being 1.35 (95% CI, 1.17-1.56), 1.22 (95% CI, 1.09-1.37), and 0.98 (95% CI, 0.84-1.14) for those who had stopped smoking for <5, 5 to 14, and 15 or more years, respectively (*P* <. 001 for trend). Similarly, for lung cancer, these RRs were 2.55 (95% CI, 1.92-3.38), 1.55 (95% CI, 1.18-2.04), and 1.11 (95% CI, 0.77-1.61), respectively (*P* <. 001 for trend). Among exsmokers, the numbers of other specific cancers were too small for separate analyses. Supporting Figure 3 (see online supporting information) shows similar results for exsmokers who had stopped because of ill health. Among them, the overall risk of all cancer (RR, 1.38; 95% CI, 1.29-1.47) was similar to that for current smokers but significantly greater than that for exsmokers who had stopped by choice, and there were significant residual risks approximately 15 years after quitting. Similar findings were also seen for lung cancer.

Among women, 8525 developed cancer between the ages of 40 and 79 years during 2.15 million person-years of follow-up. Among the very few who ever smoked regularly, the overall cancer risk was again significantly elevated (Table 1), with an adjusted RR of 1.42 (95% CI, 1.28-1.57), and it was greater, though nonsignificantly so, in urban areas (RR, 1.51; 95% CI, 1.30-1.75) than in rural areas (RR, 1.35; 95% CI, 1.17-1.56). For lung cancer, the RR was 2.28 (95% CI, 1.84-2.81); again, it was greater in urban areas (RR, 2.97; 95% CI, 2.25-3.92) than in rural areas (RR, 1.64; 95% CI, 1.18-2.26). If these associations with smoking are largely causal, then approximately 2% of all female cancers between the ages of 40 and 79 years in the current study can be attributed directly to ever regular smoking.

We applied the population attributed fractions of ever regular smoking in Table 1 to independent site-specific numbers of cancers at the ages of 35 to 69, 70 to 79, and 80+ years in mainland China, which were estimated on the basis of the incidence-to-mortality ratio.^27^–^29^ In 2010, it was estimated that smoking caused approximately 435,000 new cancer cases (and approximately 340,000 cancer deaths) in China, including 360,700 among men and 73,600 among women, with approximately 230,000 from lung cancer (203,000 in men and 28,000 in women).

## DISCUSSION

This large nationwide, prospective study provides new evidence for smoking-related cancer risks in China for the current decade. Overall, it indicates that smoking causes approximately 0.45 million cancer cases per year and accounts for approximately a quarter of all adult male cancers. These estimates chiefly reflect the consequences of past smoking patterns, and the epidemic is likely to develop further. Among urban male smokers who started before the age of 20 years (ie, the uptake pattern now typical among young men throughout China), the tobacco-attributed cancer risk is already very appreciable, and this foreshadows greater future risks for Chinese men as a whole. It is encouraging that female smoking rates remain low, and some of the male smokers in the study quit before they had developed illness and thus avoided much of the hazard.

During previous decades, several large studies assessed the effects of smoking on cancer and other diseases in particular Chinese populations; these almost exclusively involved people born during the first half of the 20th century and focused only on mortality.^13^–^18^ Those studies indicated that smoking accounted for only 12% to 13% of all adult male deaths in China in the 1990s, and the epidemic was more advanced in urban areas than in rural areas. For cancer, the previous estimates for China were not entirely consistent, with tobacco-attributed fractions ranging from 16% to 32%; this was probably due partly to differences in sampling schemes, areas covered, types of populations studied, and prevalences and definitions of smoking used. More recently, a few studies attempted to assess the smoking-related cancer burden in China for the current decade, but they still involved cohorts established decades ago of people born before 1950^30^ or used data from atypical regions of China (eg, Shanghai) where the epidemic is more advanced in comparison with other regions as a result of more prolonged use of manufactured cigarettes.^29^,^31^ A recent report estimated that in 2005, tobacco caused 0.5 million cancers in China, including 270,000 lung cancers.^29^ For lung cancer, that report used relative risks (5.7 for men and 5.0 for women) from published case-control studies in Shanghai^32^; these were much more extreme than those observed in the current study and all other previous large studies in mainland China.^16^–^18^

In China, cigarette consumption became widespread earlier in the cities (eg, Shanghai) than in the rural areas, mainly because of limited availability in rural areas before the 1980s. Consequently, as shown in our study, the epidemic is more advanced in urban men than rural men. However, this urban/rural difference is likely to diminish or even be reversed over the next few decades because rural men born during more recent decades not only have tended to start at the same age as their urban counterparts but also have a higher smoking prevalence and smoke cigarettes almost exclusively. Likewise, in urban areas, as older generations in which relatively few smoked cigarettes persistently from early adulthood are replaced by younger generations that have smoked cigarettes persistently since early adulthood, the overall tobacco-related cancer (and other disease) risk can be expected to increase, as indicated by the findings in the current study among urban men who started smoking before the age of 20 years.

The cancers chiefly associated with smoking in the current study were those previously known to be affected by smoking, including lung cancer, stomach cancer, liver cancer, esophageal cancer, and cancer at 5 other less common sites. The observed relative risk for each, though still much less extreme than those seen in Western populations, was generally consistent with findings from previous large studies in China.^13^–^18^,^29^–^31^ Although the data are still limited, our study provides the first prospective evidence in China that the association of lung cancer with smoking is much stronger for nonadenocarcinoma than adenocarcinoma. Although the current relative risks for lung cancer and several other cancers are quite modest in comparison with those for Western populations, the absolute risks associated with smoking are not because of their high rates among Chinese never-smokers. Indeed, the lung cancer rates among male and female never-smokers in the current study were more than 3 times those reported for 1990 US never-smokers (Supporting Fig. 4 [see online supporting information]), perhaps partly because of exposure to indoor air population from cooking and heating.^33^ Large changes in diet, lifestyle, and chronic infective processes, some favorable (eg, better nutrition, use of clean fuel for cooking and heating, and vaccination against hepatitis B virus) and some unfavorable (eg, lack of physical activity and increased consumption of animal fat), are now taking place in China, and they may substantially modify both the cancer risk in never-smokers and the future population impact of smoking on cancer and other diseases.^11^ It is possible that the way in which people smoke (eg, degree of inhalation) may also change somehow, but even among men who reported not always inhaling deeply into the lungs while smoking, the risks remain high.

In comparison with previous studies established 1 to 2 decades ago,^16^,^18^ a higher proportion of Chinese smokers have now quit smoking voluntarily, and this has allowed a prospective assessment of the benefits associated with cessation to be studied for the first time in China. Our study shows that if smokers do stop before they develop any serious illness, then much of the cancer risk can be avoided approximately 15 years after quitting. However, approximately half of the exsmokers in the current study quit only because of ill health, and among them, the risk of cancer (and other diseases) was, as would be expected, significantly higher than the risk for those who had stopped by choice. This highlights the need for effective educational programs and campaigns to promote voluntary cessation among adult smokers for more health gains.

In summary, approximately two-thirds of men in China now smoke cigarettes regularly, and the current study indicates that approximately 0.45 million cancer cases per year (out of approximately 2 million cases per year in adults) are now caused by smoking. The tobacco-related cancer risks among men are expected to increase substantially during the next few decades as a delayed effect of the recent rise in cigarette use unless there is widespread cessation among adult smokers. The first generation of men in China to experience the full extent of tobacco risks will probably be those who were born during the 1970s or 1980s and reached adulthood during the 1990s or 2000s when cigarette consumption in China was high. Encouragingly, the female smoking rate remains low, and unexpectedly (and without explanation), the proportion of women who have become smokers since early adulthood has decreased substantially over the past few decades. If this low uptake of smoking by young women continues, although the tobacco-attributed disease burden will increase substantially among Chinese men, it will decrease among Chinese women. Tobacco would then be responsible for most of the difference in life expectancy between men and women in China.

## FUNDING SUPPORT

This study was funded by the Kadoorie Foundation, Wellcome Trust, UK Medical Research Council, British Heart Foundation, Cancer Research UK, and National Natural Science Foundation of China (81390541).

## CONFLICT OF INTEREST DISCLOSURES

The authors made no disclosures.

The following are members of the China Kadoorie Biobank Collaborative Group: **International Steering Committee** (Jun-Shi Chen, Zheng-Ming Chen [principal investigator], Rory Collins, Li-Ming Li [principal investigator], and Richard Peto), **International Coordinating Center at Oxford** (Daniel Avery, Derrick Bennett, Yu-Mei Chang, Yi-Ping Chen, Zheng-Ming Chen, Robert Clarke, Huai-Dong Du, Xue-Juan Fan, Hai-Yan Gao, Simon Gilbert, Michael Holmes, Andri Iona, Rene Kerosi, Ling Kong, Om Kurmi, Garry Lancaster, Sarah Lewington, John McDonnell, Winnie Mei, Iona Millwood, Qun-Hua Nie, Jayakrishnan Radhakrishnan, Paul Ryder, Sam Sansome, Dan Schmidt, Paul Sherliker, Rajani Sohoni, Robin Walters, Jenny Wang, Lin Wang, Alex Williams, Ling Yang, and Xiao-Ming Yang), **National Coordinating Center at Beijing** (Zheng Bian, Ge Chen, Lei Guo, Yu Guo, Bing-Yang Han, Can Hou, Peng Liu, Jun Lv, Pei Pei, Shu-Zhen Qu, Yun-Long Tan, Can-Qing Yu, and Hui-Yan Zhou), and **Regional Coordinating Centers**, which include in **Qingdao** the Qingdao Center for Disease Control and Prevention (Zeng-Chang Pang, Shao-Jie Wang, Yong-Mei Liu, Ran-Ran Du, Yai-Jing Zang, Liang Cheng, Xiao-Cao Tian, and Hua Zhang) and the Licang Center for Disease Control and Prevention (Si-Lu Liu, Jun-Zheng Wang, and Wei Hou), in **Heilongjiang** the Provincial Center for Disease Control and Prevention (Ji-Yuan Yin, Ge Jiang, Shu-Mei Liu, Zhi-Gang Pang, and Xue Zhou) and the Nangang Center for Disease Control and Prevention (Li-Qiu Yang, Hui-Li Han, Hui He, Bo Yu, Yan-Jie Li, Jin Qi, Huai-Yi Mu, Qi-Nai Xu, Mei-Ling Dou, and Jiao-Jiao Ren), in **Hainan** the Provincial Center for Disease Control and Prevention (Jian-Wei Du, Shan-Qing Wang, Xi-Min Hu, Hong-Mei Wang, Jin-Yan Chen, Yan Fu, Zheng-Wang Fu, Xiao-Huan Wang, and Hua Dong) and the Meilan Center for Disease Control and Prevention (Min Weng, Xiang-Yang Zheng, Yi-Jun Li, Hui-Mei Li, and Cheng-Long Li), in **Jiangsu** the Provincial Center for Disease Control and Prevention (Ming Wu, Jin-Yi Zhou, Ran Tao, and Jie Yang) and the Suzhou Center for Disease Control and Prevention (Jie Shen, Yi-He Hu, Yan Lu, Yan Gao, Liang-Cai Ma, Ren-Xian Zhou, Ai-Yu Tang, Shuo Zhang, and Jian-Rong Jin), in **Guangxi** the Provincial Center for Disease Control and Prevention (Zhen-Zhu Tang, Na-Ying Chen, and Ying Huang) and the Liuzhou Center for Disease Control and Prevention (Ming-Qiang Li, Jin-Huai Meng, Rong Pan, Qi-Lian Jiang, Jing-Xin Qing, Wei-Yuan Zhang, Yun Liu, Liu-Ping Wei, Li-Yuan Zhou, Ning-Yu Chen, Jun Yang, and Hai-Rong Guan), in **Sichuan** the Provincial Center for Disease Control and Prevention (Xian-Ping Wu, Ning-Mei Zhang, Xiao-Fang Chen, and Xue-Feng Tang) and the Pengzhou Center for Disease Control and Prevention (Guo-Jin Luo, Jian-Guo Li, Xiao-Fang Chen, Jian Wang, Jia-Qiu Liu, and Qiang Sun), in **Gansu** the Provincial Center for Disease Control and Prevention (Peng-Fei Ge, Xiao-Lan Ren, and Cai-Xia Dong) and the Maiji Center for Disease Control and Prevention (Hui Zhang, En-Ke Mao, Xiao-Ping Wang, and Tao Wang), in **Henan** the Provincial Center for Disease Control and Prevention (Guo-Hua Liu, Bao-Yu Zhu, Gang Zhou, Shi-Xian Feng, Liang Chang, and Lei Fan) and the Huixian Center for Disease Control and Prevention (Yu-Lian Gao, Tian-You He, Li Jiang, Jian-Hua Qin, Hua-Rong Sun, Pan He, Chen Hu, Qian-Nan Lv, and Xu-Kui Zhang), in **Zhejiang** the Provincial Center for Disease Control and Prevention (Min Yu, Ru-Ying Hu, Le Fang, and Hao Wang) and the Tongxiang Center for Disease Control and Prevention (Yi-Jian Qian, Zhi-Ying Wu, Chun-Mei Wang, Kai-Xue Xie, Ling-Li Chen, Ya-Xing Pan, and Dong-Xia Pan), and in **Hunan** the Provincial Center for Disease Control and Prevention (Yue-Long Huang, Bi-Yun Chen, Dong-Hui Jin, Hui-Lin Liu, Zhong-Xi Fu, and Qiao-Hua Xu) and the Liuyang Center for Disease Control and Prevention (Xin Xu, You-Ping Xiong, Wie-Fang Jia, Xian-Zhi Li, Li-Bo Zhang, and Zhe Qiu).
