# Supplementary material for: Emerging Tobacco-Related Cancer Risks in China: A Nationwide, Prospective Study of 0.5 Million Adults
Source: Cancer. 2015 Sep 1;121(Suppl 17):3097–106. doi: 10.1002/cncr.29560 (PMC4584499; doi:10.1002/cncr.29560)

Web material contents list:

Webtable 1: Prevalence of smoking in men and women by various baseline characteristics

Webfigure 1: Smoking patterns by year of birth and area among men

Webfigure 2: Adjusted risk ratios for all cancer and lung cancer in male smokers, by area

Webtable 2: Number of cancer cases at age 40-79, adjusted risk ratios and population attributable fractions for current versus never smokers in men and women

Webfigure 3: Adjusted risk ratios for all cancer and lung cancer by years stopped smoking among male ex-smokers who had stopped because of ill health

Webfigure 4: Lung cancer incident rates, in Chinese smokers, compared with Chinese and US non-smokers

Webtable 1: Prevalence of smoking in men and women by various baseline characteristics

	No. of People	Never Smoker, %	Regular Smoker, %				
			All	Former	Current	Current cigarette	Current cigarette only
**<u>Men</u>**							
Overall	210,259	25.7	74.3	13.3	61.1	54.1	51.8
Mean exhaled CO (ppm)		*5.4*	*13.2*	*5.2*	*14.9*	*15.4*	*15.3*
**Location**							
Urban	68,994	33.6	66.4	15.9	50.5	49.3	48.6
Rural/semi-rural	141,265	21.8	78.2	12.0	66.2	56.4	53.4
**Age group (years)**							
30-39	29,601	31.5	68.5	5.6	62.9	61.6	60.4
40-49	59,240	23.7	76.3	8.7	67.6	64.6	62.6
50-59	63,725	22.2	77.8	13.6	64.2	55.8	52.9
60-69	41,339	27.0	73.0	19.7	53.2	40.5	37.9
70-79	16,354	32.0	68.0	26.3	41.7	30.0	28.1
**Highest education**							
No formal School	18,667	19.3	80.7	16.2	64.5	46.5	42.6
Primary School	70,121	20.9	79.1	14.4	64.7	53.1	50.0
Middle or high school	104,915	26.7	73.3	12.0	61.3	58.3	56.6
College or University	16,556	46.4	53.6	13.4	40.2	39.9	39.7
**Household income (yuan/year)**							
< 5,000	19,457	23.5	76.5	12.8	63.7	37.2	31.5
5,000-19,999	35,288	24.0	76.0	11.0	65.1	50.6	45.5
20,000-34,999	59,568	25.9	74.1	13.9	60.2	54.5	52.3
>=35,000	53,412	26.1	73.9	14.1	59.8	58.3	57.5
**<u>Women</u>**							
Overall	302,632	96.8	3.2	0.9	2.4	2.1	2.1
Mean exhaled CO (ppm)		*4.5*	*9.0*	*3.9*	*10.9*	*11.2*	*11.2*
**Location**							
Urban	103,932	96.4	3.6	1.0	2.6	2.3	2.3
Rural/semi-rural	198,700	96.9	3.1	0.8	2.3	2.0	2.0
**Age group (years)**							
30-39	48,203	99.3	0.7	0.1	0.7	0.7	0.7
40-49	93,508	98.6	1.4	0.2	1.2	1.2	1.2
50-59	93,831	97.1	2.9	0.7	2.2	2.1	2.0
60-69	50,434	92.5	7.5	2.4	5.1	4.4	4.4
70-79	16,656	89.9	10.1	3.8	6.3	5.2	5.1
**Highest education**							
No formal School	76,554	95.5	4.5	1.4	3.1	2.6	2.6
Primary School	95,095	95.8	4.2	1.2	3.1	2.8	2.8
Middle or high school	117,525	98.1	1.9	0.4	1.5	1.5	1.5
College or University	13,458	99.0	1.0	0.2	0.8	0.8	0.7
**Household income (yuan/year)**							
< 5,000	30,746	91.8	8.2	2.4	5.7	4.7	4.6
5,000-19,999	59,341	95.9	4.1	1.1	3.1	2.8	2.7
20,000-34,999	89,445	96.8	3.2	0.8	2.3	2.2	2.2
>=35,000	73,309	98.2	1.8	0.5	1.3	1.3	1.3

Webfigure 1. Smoking patterns by year of birth & area among men

(white square: rural, black square: urban)

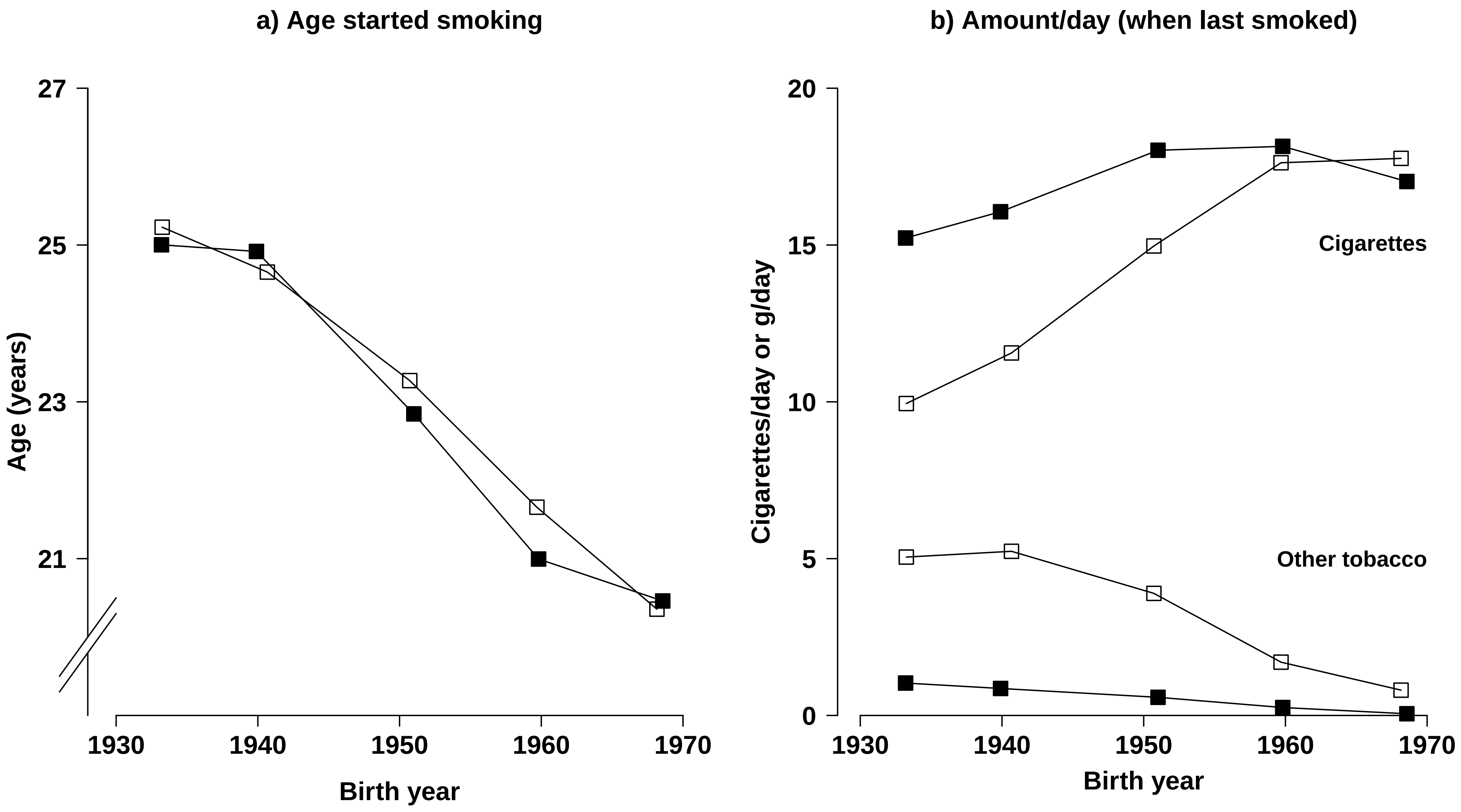

Webfigure 2: Adjusted risk ratios for all cancer and lung cancer in male smokers, by area

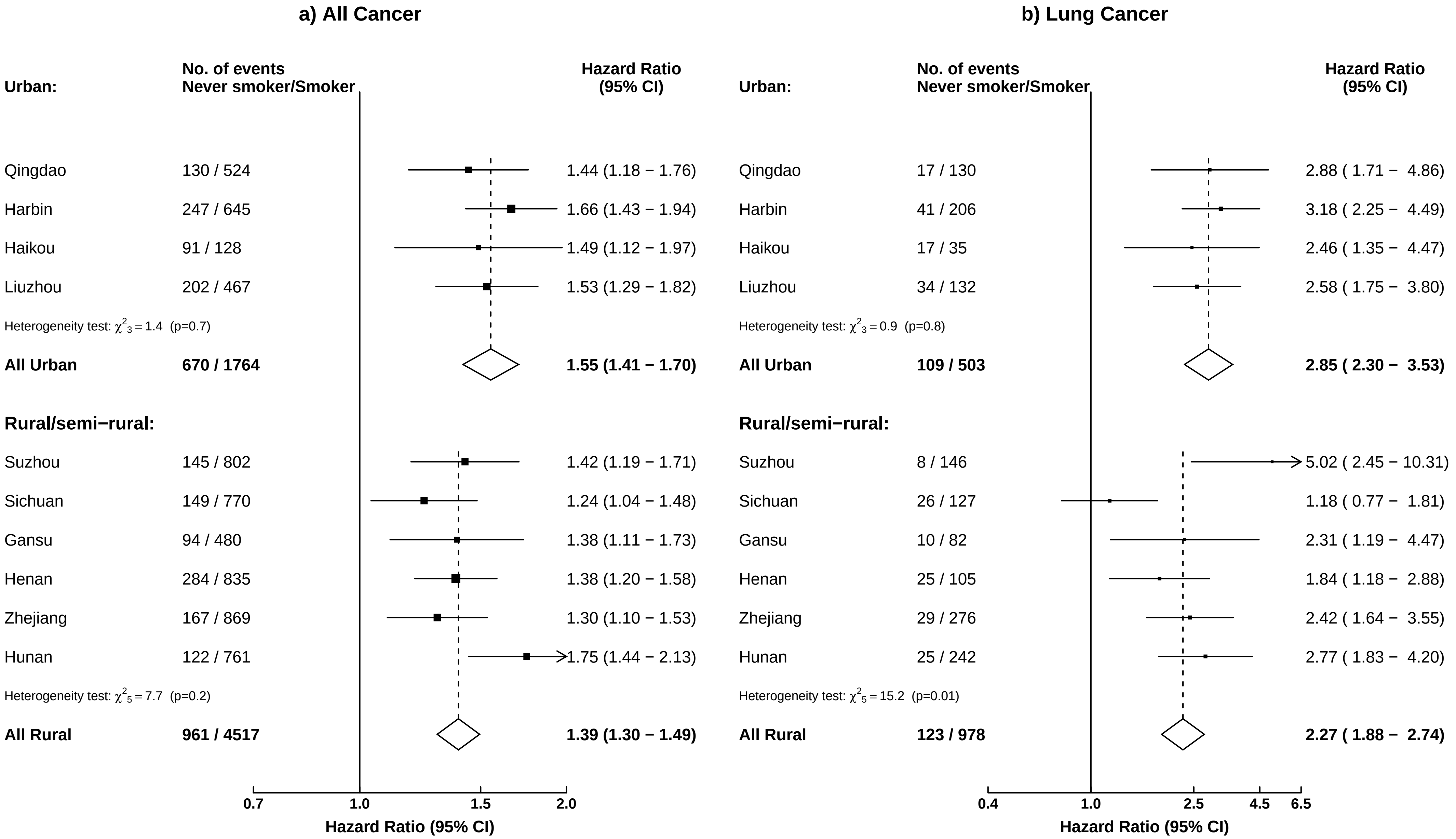

Webtable 2: Number of cancer cases at age 40-79, adjusted risk ratios and population attributable fractions for current versus never smokers in men and women[§]

	Men				Women			
	No. of Events				No. of Events			
	Never Smokers	Current Smokers[a]	RR (95% CI)[b]	PAF (%)[c]	Never Smokers	Current Smokers[a]	RR (95% CI)[b]	PAF (%)[c]
Lung cancer	232	1290	2.63 (2.28-3.04)	43	1031	99	2.26 (1.80-2.83)	5
Liver cancer	207	662	1.28 (1.09-1.51)	14	458	25	1.34 (0.87-2.08)	1
Stomach cancer	250	812	1.33 (1.15-1.55)	16	582	28	1.26 (0.84-1.90)	1
Oesophagus cancer	179	694	1.55 (1.31-1.83)	24	442	12	1.12 (0.60-2.10)	0
Breast cancer					1286	28	0.97 (0.66-1.44)	0
Five minor sites[d]	131	450	1.53 (1.25-1.87)	22	421	18	1.11 (0.67-1.82)	0
All other cancers	632	1507	1.09 (0.98-1.20)	4	3786	147	1.25 (1.05-1.49)	1
**All cancer**	1631	5415	1.46 (1.37-1.54)	20	8006	357	1.38 (1.24-1.55)	1

[§] Excludes 2577 participants (969 male) with cancer at baseline.

[a] Excludes ever regular smokes who had stopped smoking by choice or because of ill health.

[b] Risk ratios were adjusted for area and age at risk (strata) , and education (4-levels) and alcohol (never, occasional, regular). Confidence intervals (CI) are for the comparison of current smokers vs. never smokers.

[c] RR used for calculating PAF is for current smokers.

[d] Five minor sites: Mouth, Pharynx, Bladder, Larynx or Pancreas Cancer (ICD-10: C00-C14, C25, C32 or C67).

Webfigure 3: Adjusted risk ratios for all cancer and lung cancer by years stopped smoking among male ex−smokers who had stopped because of ill health

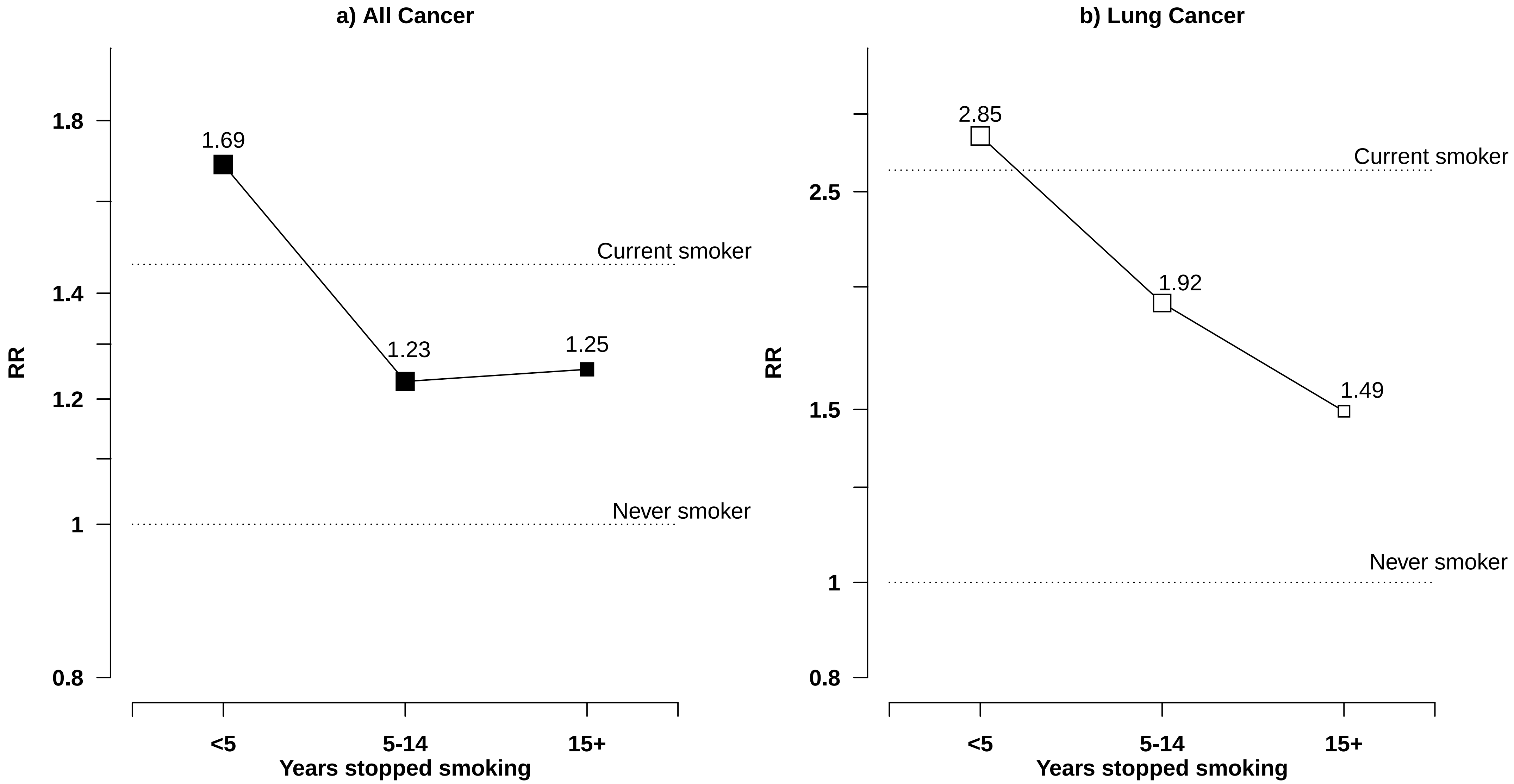

**Years stopped smoking (mean):**	<5 (2.4)	5−14 (8.5)	15+ (21.2)
**No. of events:**	355	332	179
**RR (95% CI):**	1.69 (1.52−1.87)	1.23 (1.05−1.37)	1.25 (1.08−1.45)

**Years stopped smoking (mean):**	<5 (2.4)	5−14 (8.5)	15+ (21.2)
**No. of events:**	85	74	32
**RR (95% CI):**	2.85 (2.29−3.54)	1.92 (1.53−2.42)	1.49 (1.05−2.12)

Webfigure 4: Lung cancer incidence rates, in Chinese smokers compared with Chinese and US never smokers

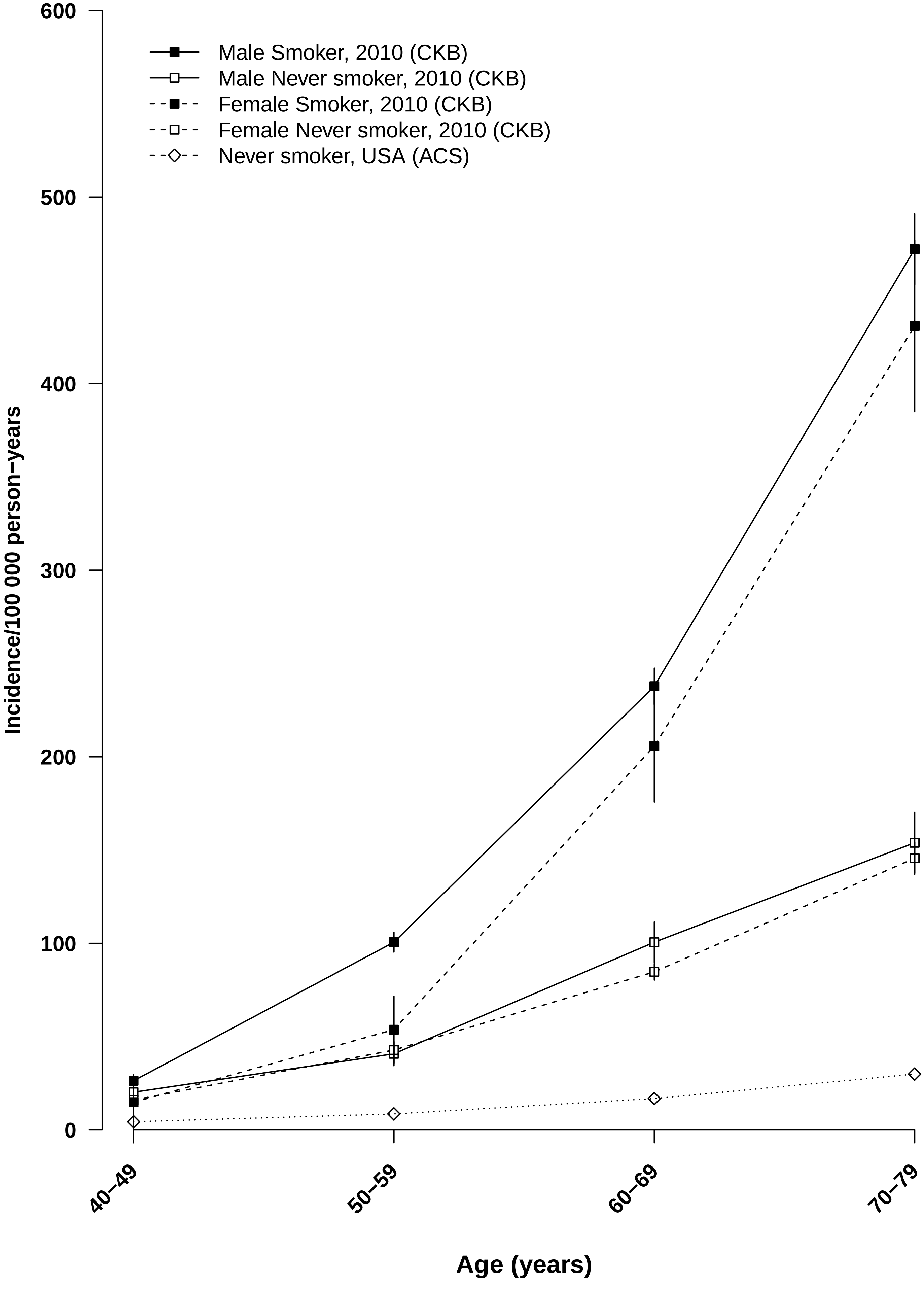

Supplement: Supplementary file 1 — Supplementary Information [file cncr0121-3097-sd1.pdf]
